# Molecular Characterization of Enterohemorrhagic *E. coli* O157 Isolated from Animal Fecal and Food Samples in Eastern China

**DOI:** 10.1155/2014/946394

**Published:** 2014-06-04

**Authors:** Shaohui Wang, Shuxiao Zhang, Zhe Liu, Pingping Liu, Zixue Shi, Jianchao Wei, Donghua Shao, Beibei Li, Zhiyong Ma

**Affiliations:** Shanghai Veterinary Research Institute, Chinese Academy of Agricultural Sciences, Shanghai 200241, China

## Abstract

*Objective*. To elucidate the extent of food contamination by enterohemorrhagic *Escherichia coli* (EHEC) O157 in Eastern China. *Methods*. A total of 1100 food and animal fecal samples were screened for EHEC O157. Then, molecular characterization of each isolate was determined. *Results*. EHEC O157 was isolated as follows: pig feces, 4% (20/500); cattle feces, 3.3% (2/60); chicken feces, 1.43% (2/140); pork, 2.14% (3/140), milk, 1.67% (1/60); and chicken meat, 1.67% (1/60). The *stx1*, *stx2*, *eae*, and *hlyA* genes were present in 26.7% (8/30), 40% (12/30), 63.3% (19/30), and 50% (15/30) of the O157 isolates, respectively. Molecular typing showed that strains from fecal and food samples were clustered into the same molecular typing group. Furthermore, the isolates from pork and pig feces possessed the same characterization as the clinical strains ATCC35150 and ATCC43889. Biofilm formation assays showed that 53.3% of the EHEC O157 isolates could produce biofilm. However, composite analyses showed that biofilm formation of EHEC O157 was independent of genetic background. *Conclusions*. Animal feces, especially from pigs, serve as reservoirs for food contamination by EHEC O157. Thus, it is important to control contamination by EHEC O157 on farms and in abattoirs to reduce the incidence of foodborne infections in humans.

## 1. Introduction

Enterohemorrhagic* Escherichia coli *(EHEC) is an important emerging zoonotic foodborne pathogen that can cause watery and/or bloody diarrhea, hemorrhagic colitis (HC), hemolytic-uremic syndrome (HUS), and thrombotic thrombocytopenic purpura (TTP). In humans, EHEC O157 is recognized as a major etiological agent of these diseases [1–3]. Shiga toxin, intimin, and enterohemolysin are key virulence factors for the pathogenesis of O157 and other EHEC strains, including the 2011 O104:H4 outbreak in Germany [1, 4, 5]. Biofilm is composed of surface-bound microbes enclosed in an amorphous extracellular matrix [6], which is often an assembly of exopolysaccharides, proteins, and nucleic acids [7]. Residence in a biofilm community offers certain advantages to bacteria, such as enhanced resistance to the environmental stresses.

Food contamination has been identified as a potential source of pathogenic EHEC O157 transmission in humans [8, 9]. Furthermore, the major animal reservoirs of EHEC O157 are primarily cattle, followed by sheep, goats, pigs, and chickens [10, 11]. Molecular typing methods, such as random-amplified polymorphic DNA (RAPD) fingerprinting, multilocus sequence typing (MLST), and phylogenetic typing, are useful in determining the relatedness of pathogens in disease outbreak investigations by public health laboratories [12–18].

Although the prevalence of EHEC O157 in animal feces in Eastern China has been reported [11], comparative analysis of EHEC O157 in animal feces and food samples remains limited. Thus, the aim of this study was to detect and characterize EHEC O157 isolates from animal fecal and food samples in Eastern China to elucidate the extent of food contamination by animal feces.

## 2. Materials and Methods

### 2.1. Bacterial Strains and Growth Conditions

The EHEC O157:H7 reference strains ATCC35150 and ATCC43889 were used as positive controls for amplification by polymerase chain reaction (PCR) and molecular typing. All* E. coli* cells were routinely grown in Luria-Bertani (LB) broth at 37°C with aeration and stored at −80°C in 20% glycerol for further use.

### 2.2. Isolation and Identification of EHEC O157

In the present study, a total of 1100 samples, including animal fecal samples (*n* = 800) and food samples (*n* = 300), which were collected once per month from July 2010 to July 2012 in Eastern China (Shanghai, Jiangsu, and Zhejiang), were used for analysis. Isolation and identification of EHEC O157 were performed according to a procedure using enrichment methods and confirmed by PCR. Briefly, preliminary identification of O157 strains was obtained through phenotype characterization using sorbitol-MacConkey agar. Isolates were confirmed by PCR for the O157 lipopolysaccharide O-antigen synthesis gene* rfbE* (Table 1) and serotyping was performed using an agglutination assay with anti-O157 sera.

### 2.3. Virulence Genes Detection

The EHEC O157 virulence genes* stx1, stx2*,* hlyA*, and* eae* were assessed by PCR. Descriptions of the targeted genes and primer sequences are listed in Table 1.

### 2.4. Quantification of Biofilm Formation

Biofilm formation assays were performed according to the methods described in a previous report [21] with some modifications. Briefly, strains were grown to the stationary phase in LB broth at 37°C and then diluted to 1 : 100 in LB broth without salt (LB-NS). A 200 *μ*L aliquot of each dilution was dispensed into individual wells of a microtiter plate. Wells with sterile LB medium served as blank controls. After incubation for 24 h at 37°C, the cultured medium was removed and the wells were washed twice with sterile phosphate-buffered saline (PBS) to remove planktonic bacteria. The biofilms grown in the microtiter plate were stained with crystal violet (1%, w/v) at room temperature for 1 h. After removing the crystal violet solution, the wells were washed five times with PBS to remove unbound dye. Then, the plates were air dried and filled with 200 *μ*L of 95% ethanol to solubilize the dye from the cells. Finally, the optical density (OD) of the stained adherent bacteria was determined at 595 nm using a microplate reader (BioTek Instruments, Inc., Winooski, VT, USA). All tests were performed three times and the results were averaged.

Strains were classified as non-biofilm producers, weak biofilm producers, moderate biofilm producers, or strong biofilm producers based on OD_595_ measurements of bacterial biofilms in accordance with previously described criteria [22]. Briefly, OD values produced by bacterial biofilms were compared to a cut-off OD value (ODc, three standard deviations above the mean OD of a blank test) and the strains were classified as follows: no biofilm production (OD < ODc), weak biofilm producer (ODc < OD < 2 ODc), moderate biofilm producer (2 ODc < OD < 4 ODc), and strong biofilm producer (4 ODc < OD).

### 2.5. Phylogenetic Typing

*E. coli *is classified to four main phylogenetic groups (A, B1, B2, and D) according to the combination of two genes (*chuA *and* yjaA*) and an anonymous DNA fragment (TSPE4.C2) as described previously [22]. Thus, EHEC O157 isolates were assigned to phylogenetic groups by triplex-PCR amplification of* chuA* and* yjaA*, and the DNA fragment TSPE4.C2.

### 2.6. Multilocus Sequence Typing (MLST)

All EHEC O157 strains were assigned to multilocus sequence types (STs) as described previously [23] for the amplification of seven house-keeping genes (*adk*,* fumC*,* gyrB*,* icd*,* mdh*,* purA*, and* recA*). Briefly, the housekeeping genes of each strain were amplified, sequenced, and aligned against allele templates of* E. coli *retrieved from an online database (http://www.mlst.net/).

### 2.7. Random-Amplified Polymorphic DNA (RAPD) Fingerprinting

Based on the results obtained in preliminary experiments, only one primer, RAPD-2 (5′-TGCCCAGCCT-3′), was used for RAPD-PCR analysis of the EHEC O157 strains. Amplification reactions were performed in a 20 *μ*L volume containing 10 *μ*L of 2 × PCR Master Mix, 1.0 *μ*L of primer (10 *μ*M), 1.0 *μ*L of EHEC O157 DNA, and 8 *μ*L of sterile ddH_2_O. The RAPD-PCR reaction comprised the following steps: predenaturation at 95°C for 2 min, 35 cycles at 95°C for 30 s, 36°C for 30 s, and 72°C for 2 min, followed by a final elongation step at 72°C for 10 min.

## 3. Results

### 3.1. Prevalence of EHEC O157

To compare the prevalence of EHEC O157 in animal fecal and food samples, 1100 isolates were characterized using selective media, PCR, and an anti-O157 sera agglutination assay. Of these, 30 (2.73%) EHEC O157 strains were successfully isolated from the obtained samples. As shown in Table 2, the proportion of EHEC O157 isolates was higher in animal feces (3.13%, 25/800) than food samples (1.67%, 5/300). In fecal samples, EHEC O157 was detected in 4% (20/500) of the pig samples, 3.3% (2/60) of the cattle samples, 1.43% (2/140) of the chicken samples, and 1% (1/100) of the duck samples. The prevalence of EHEC O157 in pork was 2.14% (3/140) and 1.67% (1/60) in both milk and chicken, respectively.

### 3.2. Distribution of Virulence Genes in EHEC O157

EHEC O157 isolates were screened for the presence of virulence genes. The distribution and combinations of virulence genes for each isolate are shown in Table 3. Ten virulence gene genotypes (G1–G10) were identified. The* stx1* and* stx2* genes were detected in 8 (26.7%) and 12 (40%) isolates, respectively. The* eae* and* hlyA* genes were present in 63.3% (19/30) and 50% (15/30) of the O157 isolates, respectively. Moreover, 7 (23.3%) isolates harbored the* eae* gene alone, which was the predominant genotype (G1). Five (16.7%) strains harbored all four virulence genes simultaneously (G3), which were all isolated from pig feces and pork samples. Generally, the prevalence of these genes in pig feces and pork was higher than in other samples.

### 3.3. Biofilm Formation

**Figure 1:**
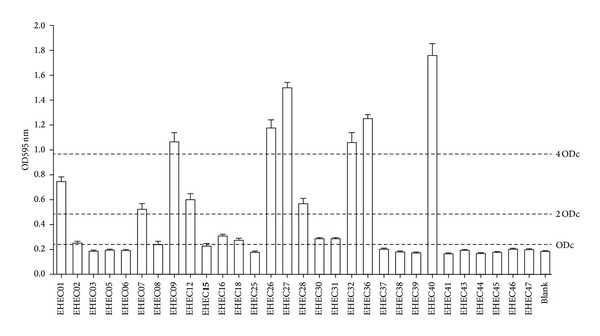
Biofilm formation by EHEC O157 isolates. Biofilm formation by EHEC O157 isolates on polypropylene microtiter plates following incubation for 24 h at 37°C in LB broth without salt (LB-NS). All experiments were repeated at least three times. The columns represent the mean ± standard deviations of the data. Comparisons of the OD values produced by bacterial biofilms to the ODc values were used to classify the strains (non-biofilm producer, weak, moderate, or strong). The cut-off OD (ODc) value was defined as three standard deviations above the mean OD of a blank control. Strains were classified as follows: OD < ODc = no biofilm production; ODc < OD < (2 ODc) = weak biofilm producer; (2 ODc) < OD < (4 ODc) = moderate biofilm producer; and (4 ODc) < OD = strong biofilm producer.

The ability of EHEC O157 isolates to form biofilm in polystyrene microtiter plates was assessed according to OD_595_ values. The results indicated that biofilm formation occurred in 16 (53.3%) of the EHEC O157 strains after a 24 h incubation at 37°C (Figure 1, Table 3). Among these strains, 20% (6/30) were strong biofilm producers, 13.3% (4/30) were moderate biofilm producers, and 20% (6/30) were weak biofilm producers, respectively.

### 3.4. Molecular Typing of EHEC O157

Of the individual typing techniques used in this study, phylogenetic typing showed that most of the O157 isolates belonged to group D (70%). Only 16.7% (5/30) of the strains belonged to group A, 10% (3/30) to B2, and 3.3% (1/30) to B1 (Table 3). Thus, EHEC O157 was strongly associated with phylogenetic group D.

Among 30 EHEC O157 isolates analyzed by MLST, 27 strains generated sequence tracings acceptable for an ST number available from the online MLST database (http://www.mlst.net/). Overall, the seven-gene MLST scheme defined nine sequence types, with one ST (ST 11) encompassing 33.3% (10/30) of the EHEC O157 isolates in this study, which were the same as the reference strains ATCC35150 and ATCC43889. The second most common sequence type was ST 88, which accounted for 16.7% (5/30) of the isolates, followed by ST 641 (13.3%, 4/30) and ST 117 (10%, 3/30) (Table 3).

RAPD analysis showed that the isolates formed 10 RAPD types (R1–R10) with bands ranging from approximately 200 to 2100 bp. A total of 9 (30%) EHEC O157 isolates were grouped into RAPD type R1. RAPD types R2, R3, and R4 were composed of 7 (23.3%), 4 (13.3%), and 4 (13.3%) isolates, respectively. The other RAPD types, R5–10, were composed of a single isolate. The predominant RAPD types were EHEC O157 from pig feces and pork samples (Table 3).

## 4. Discussion

EHEC O157 is an important emerging zoonotic foodborne pathogen that can cause gastroenteritis often complicated by HC or HUS in humans [1–3]. Food contaminated with EHEC O157 is a principal source of infection in humans [8, 9]. Thus, the aim of this study was to isolate and characterize EHEC O157 strains from animal feces and food samples to elucidate the extent of food contaminated by animal feces in Eastern China.

In the present study, EHEC O157 was successfully isolated from 2.73% (30/1100) of the tested animal feces and food samples. Among the 30 EHEC O157 isolates, 20 (66.7%) strains were isolated from pig feces, 3 (10%) from pork, 2 (6.67%) from cattle feces, 2 (6.67%) from chicken feces, and one each from duck feces, milk, and chicken meat, respectively. Reportedly, the proportion of EHEC O157 is often higher in pig feces and pork than in cattle feces and beef [24]. Although the number of EHEC O157 isolates in pig feces or pork was higher than in cattle feces or milk, the prevalence was similar in our investigation. Two (3.3%) of the 60 cattle fecal samples were positive for EHEC O157, but none of the beef samples were. One possible explanation for this phenomenon might be that the cattle fecal samples were collected from dairy cows used for milk production. The prevalence of EHEC O157 is dependent on various factors, including the hygienic conditions of the farms and abattoirs. Also, surrounding environments can impact the distribution of EHEC O157 in animal feces, food products, and water sources worldwide [8, 10, 11, 25–27]. Similarly, the prevalence of EHEC O157 from different sources in different areas of China can also extensively vary [11, 28–30]. Compared to the findings of previous studies [29], the prevalence of EHEC O157 in pig and cattle feces were lower in Shanghai, which was likely due to improved hygienic conditions of farms in recent years. Moreover, our results showed that the proportion of EHEC O157 was higher in feces than in food and water samples, which was in accordance with the results of previous studies [10, 25, 26, 29, 31].

The pathogenesis of EHEC O157 is associated with several virulence factors, such as Shiga toxin 1 and 2 (encoded by* stx1* and* stx2*, resp.), intimin (encoded by* eae*), and enterohemolysin (encoded by* hlyA*) [1, 4]. Shiga toxins inhibit protein synthesis resulting in the death of host cells and are key virulence factors of other Shiga toxin-producing* E. coli* infections, including the recent O104:H4 outbreak in Germany in 2011 [5]. Intimin is a type III secretion system effector protein that facilitates the intimate adherence of* E. coli* O157 cells to the intestinal epithelium. Enterohemolysin has been implicated as a causative agent of enterocyte effacement [1, 4]. Thus, the distribution of virulence genes in EHEC O157 isolates was screened by PCR analysis. Our results showed that more of the isolates harbored the* stx2* gene than* stx1* (40% versus 26.7%, resp.). A large proportion (63.3%) of the O157 isolates was positive for the* eae* gene, and 15 (50%) isolates harbored the* hlyA* gene. However, different distributions of these four virulence genes in EHECO157 were observed by other researchers [25, 32], which might be due to the different sources and pathogenesis of* E. coli *collections. Composite analysis showed that these virulence genes were mostly prevalent in isolates from pig feces and pork than other samples. Similar results were also reported previously [31]. Among the genotypes, 5 (16.7%) isolates from pig feces and pork possessed a combination of all four virulence genes found in clinic strains ATCC35150 and ATCC43889.

Our findings indicated that the use of molecular typing provides valuable information that may also be useful in pinpointing sources of food contamination [14, 16, 33]. In the present study, three different methods, including phylogenetic typing, MLST, and RAPD fingerprinting, were used to analyze the relatedness of EHEC O157 strains isolated from fecal and food samples. Of the individual typing techniques used in this study, MLST and RAPD fingerprinting showed higher levels of discriminating power than phylogenetic typing. Moreover, the results of the MLST and RAPD fingerprinting techniques were similar. Results of phylogenetic typing showed that most (70%) of the O157 strains were classified as group D. Parallel findings, in which EHEC O157:H7 strains were almost exclusively group D, have been reported elsewhere [17, 18]. In this study, seven selected housekeeping genes were chosen to assess potential sequence diversity by MLST typing according to a protocol found at http://www.mlst.net/. The results showed that O157 isolates predominantly belonged to types ST11, ST88, ST641, and ST117. However, different ST types were obtained in other studies, which were due to the selection of different housekeeping and virulence genes [3, 14–16].

Composite analysis of all three molecular typing methods revealed a relationship between the phylogenetic group and MLST and RAPD typing. For example, the largest cluster of MLST type (ST11) was comprised mainly of strains belonging to ECOR group D and RAPD type R1. In fact, all of the R1 type isolates were identified as ST11 and ECOR group D. Molecular characterization of EHEC O157 showed that the isolates from fecal and food samples were clustered into the same predominant group, indicating that animal feces might be a reservoir for EHEC O157. Thus, close monitoring of possible food contamination by EHEC O157 should be reinforced.

Biofilm formation aids in the survival of bacteria and enhances their ability to survival in environment. Thus, the ability of the EHEC O157 isolates to form biofilms was assessed in this study. The results indicated that biofilm formation occurred in 16 (53.3%) of the EHEC O157 strains after 24 h of incubation at 37°C. However, the composite analysis between molecular typing and biofilm formation revealed that biofilm formation of EHEC O157 was independent of the three molecular genetic typing methods used in this study, which was consistent with the conclusions of another study [17].

## 5. Conclusions

EHEC O157 was isolated from fecal and food samples and then characterized. The results showed that isolates from fecal and food samples harbored the same gene combinations. Moreover, these isolates were clustered into the same molecular typing group, indicating that animal feces are reservoirs of EHEC O157. Thus, it is important to control food contamination with EHEC O157 on farms and in abattoirs to reduce the incidence of foodborne infections in humans.

## Figures and Tables

**Table 1:** Primers for detection of virulence genes in EHEC O157 isolates.

Primers	Sequence (5′→3′)	Annealing temperature/°C	Product size/bp	Reference
*rfbE*O157-F	AAGATTGCGCTGAAGCCTTTG	55	497	[19]
*rfbE*O157-R	CATTGGCATCGTGTGGACAG			
*stx1*-F	TGTCGCATAGTGGAACCTCA	53	655	[20]
*stx1*-R	TGCGCACTGAGAAGAAGAGA			
*stx2*-F	CCATGACAACGGACAGCAGTT	53	780	This study
*stx2*-R	CCTGTCAACTGAGCACTTTG			
*eae*-F	GGTGAAACTGTTGCCGATCT	50	1382	This study
*eae*-R	TTGCCATTACGGTCATAGGCG			
*hlyA*-F	ACGATGTGGTTTATTCTGGA	50	167	This study
*hlyA*-R	CTTCACGTCACCATACATAT			

**Table 2:** Number of *E. coli* O157 identified from samples obtained from different sources.

Source	Number of samples	Number of* E. coli *O157	Percentage
**Feces**	**800**	**25**	**3.13%**
Pigs	500	20	4%
Cattle	60	2	3.3%
Chicken	140	2	1.43%
Duck	100	1	1%
**Foods**	**300**	**5**	**1.67%**
Pork	140	3	2.14%
Beef	20	0	0
Milk	60	1	1.67%
Chicken	60	1	1.67%
Duck	20	0	0
**Total**	**1100**	**30**	**2.73%**

**Table 3:** Genotypic and phenotypic characteristics of EHEC O157 isolates.

Strains	Virulence genes					MLST	Phenotypic group	RAPD type	Biofilm formation	Source
	*stx1 *	*stx2 *	*hlyA *	*eae *	Gene combinations					
ATCC35150	+	+	+	+	G3	ST11	D	R1	No	ATCC
ATCC43889	+	+	+	+	G3	ST11	D	R1	No	ATCC
EHEC06	−	−	+	−	G2	ST11	D	R1	No	Pig feces
EHEC07	+	+	+	+	G3	ST11	D	R1	Moderate	Pork
EHEC31	+	−	−	−	G5	ST11	D	R1	Weak	Pig feces
EHEC32	+	−	−	−	G5	ST11	D	R1	Strong	Pig feces
EHEC36	−	+	−	−	G6	ST11	D	R1	Strong	Pork
EHEC37	+	+	+	+	G3	ST11	D	R1	No	Pig feces
EHEC38	+	+	+	+	G3	ST11	D	R1	No	Pig feces
EHEC39	+	+	+	+	G3	ST11	D	R1	No	Pig feces
EHEC41	+	+	+	+	G3	ST11	D	R1	No	Pig feces
EHEC40	−	+	+	+	G4	ST11	D	R9	Strong	Pig feces
EHEC05	−	+	−	+	G7	ST88	A	R2	No	Pig feces
EHEC46	−	+	−	−	G6	ST88	A	R2	No	Pig feces
EHEC47	−	+	−	+	G7	ST88	A	R2	No	Pig feces
EHEC09	−	−	+	−	G2	ST88	D	R2	Strong	Chicken feces
EHEC12	−	−	+	−	G2	ST88	D	R2	Moderate	Chicken
EHEC03	−	−	+	−	G2	ST641	D	R3	No	Chicken feces
EHEC25	−	−	+	−	G2	ST641	D	R3	No	Pork
EHEC43	−	−	−	+	G1	ST641	D	R3	No	Pig feces
EHEC45	−	−	−	+	G1	ST641	D	R3	No	Pig feces
EHEC16	−	−	−	+	G1	ST117	D	R4	Weak	Cattle feces
EHEC18	−	+	+	+	G4	ST117	D	R4	Weak	Pig feces
EHEC30	−	−	−	+	G1	ST117	D	R4	Weak	Milk
EHEC44	−	−	−	+	G1	ST10	A	R10	No	Pig feces
EHEC02	+	−	−	+	G8	ST224	D	R2	Weak	Pig feces
EHEC15	−	−	+	−	G2	ST278	B1	R2	No	Cattle feces
EHEC28	−	−	−	−	G9	ST1114	B2	R4	Moderate	Pig feces
EHEC27	−	−	−	+	G1	ST1602	A	R8	Strong	Pig feces
EHEC01	−	−	+	+	G19	Unknown	B2	R5	Moderate	Pig feces
EHEC08	−	+	+	+	G4	Unknown	B2	R6	Weak	Duck feces
EHEC26	−	−	−	+	G1	Unknown	D	R7	Strong	Pig feces
